# Do attention-deficit/hyperactivity symptoms influence treatment outcome in gambling disorder?

**DOI:** 10.1192/j.eurpsy.2023.212

**Published:** 2023-07-19

**Authors:** C. Vintró-Alcaraz, G. Mestre-Bach, R. Granero, M. Gómez-Peña, L. Moragas, F. Fernández-Aranda, M. N. Potenza, S. Jiménez-Murcia

**Affiliations:** 1 Psychiatry, Hospital Universitari de Bellvitge, L’Hospitalet de Llobregat; 2 Ciber Fisiopatología Obesidad y Nutrición (CIBERObn), Instituto de Salud Carlos III, Madrid; 3 Psychiatry and Mental Health Group, Neuroscience Program, Institut d’Investigació Biomèdica de Bellvitge - IDIBELL, L’Hospitalet de Llobregat; 4 Universidad Internacional de La Rioja, La Rioja; 5 Psicobiologia i Metodologia de les Ciències de la Salut, Universitat Autònoma de Barcelona, Barcelona, Spain; 6 Yale University School of Medicine, New Haven, United States

## Abstract

**Introduction:**

Numerous studies point to the comorbidity between gambling disorder (GD) and attention deficit hyperactivity disorder (ADHD). However, there is a lack of research exploring how ADHD symptoms might influence psychological treatment outcomes for GD.

**Objectives:**

Therefore, we aimed to explore differences between patients with GD with and without ADHD symptoms regarding psychopathology, personality, sociodemographic and especially treatment outcome measures.

**Methods:**

This longitudinal study included *n*=170 patients with GD receiving 16 sessions of cognitive behavioral therapy (CBT) in a specialized unit of a public hospital. Multiple self-reported instruments were used to assess GD severity, personality, ADHD and other symptoms and sociodemographic measures prior to treatment.

**Results:**

A clinical profile characterized by greater GD severity, higher psychopathology and impulsivity, and less adaptive personality features was observed in patients with self-reported ADHD symptoms compared to those without. No significant differences in treatment response (measured by dropout and relapse rates) were reported between the two groups. However, patients with ADHD symptoms described more severe relapses (more money gambled) and GD patients who relapsed scored higher on measures of ADHD, particularly inattention.

**Conclusions:**

Individuals with GD and ADHD may experience more severe relapses following treatment, suggesting a need for more vigilant follow-up and interventions for patients with this comorbidity.

**Disclosure of Interest:**

C. Vintró-Alcaraz: None Declared, G. Mestre-Bach: None Declared, R. Granero: None Declared, M. Gómez-Peña: None Declared, L. Moragas: None Declared, F. Fernández-Aranda Consultant of: Novo Nordisk and editorial honoraria as EIC from Wiley, M. Potenza Consultant of: Opiant Pharmaceuticals, Idorsia Pharmaceuticals, AXA, Game Day Data, Baria-Tek and the Addiction Policy Forum; has been involved in a patent application with Yale University and Novartis; has received research support (to Yale) from Mohegan Sun Casino and Connecticut Council on Problem Gambling; and has consulted for and/or advised gambling and legal entities on issues related to impulse-control/addictive disorders, S. Jiménez-Murcia: None Declared

